# Variants in genes related to inflammation and endothelial function can increase the risk for carotid atherosclerosis in southwestern China

**DOI:** 10.3389/fneur.2023.1174425

**Published:** 2023-05-24

**Authors:** Yong Xie, Ming Yu, Ting Qing, Hua Luo, Minjie Shao, Wei Wei, Xingyang Yi

**Affiliations:** ^1^Department of Neurology, The People’s Hospital of Deyang City, Deyang, Sichuan, China; ^2^Department of Neurology, The Suining Central Hospital, Suining, Sichuan, China; ^3^Department of Neurology, The Second People’s Hospital of Deyang City, Deyang, China; ^4^Department of Neurology, The Affiliated Hospital of Southwest Medical University, Luzhou, Sichuan, China; ^5^Department of Neurology, The First People’s Hospital of Wenling, Wenling, Zhejiang, China

**Keywords:** high risk stroke population, carotid atherosclerosis, inflammation, endothelial function, genetic polymorphism

## Abstract

**Aim:**

To investigate the potential association between polymorphisms in genes involved in endothelial function, inflammation and carotid atherosclerosis.

**Methods:**

This was a three-center, population-based sectional survey conducted in Sichuan province of southwestern China. We randomly selected 8 different communities in Sichuan, and the residents in each community volunteered to participate in the survey by face-to-face questionnaire. A total of 2,377 residents with high stroke risk population in the 8 communities were included. Carotid atherosclerosis was evaluated by carotid ultrasound, and the 19 single nucleotide polymorphisms (SNPs) in 10 endothelial function as well as inflammation relevant genes were measured in the high stroke risk population. Carotid atherosclerosis was defined by the presence of carotid plaque or any carotid stenosis ≥15% or mean intima-media thickness (IMT) > 0.9 mm. Generalized multifactor dimensionality reduction (GMDR) approach was used to analyze gene–gene interactions among the 19 SNPs.

**Results:**

Among the 2,377 subjects with high stroke risk, 1,028 subjects had carotid atherosclerosis (43.2%), of which 852 (35.8%) cases had carotid plaque, 295 (12.4%) cases had ≥15% carotid stenosis, whereas 445 (18.7%) had mean IMT > 0.9 mm. Multivariate logistic regression revealed that *IL1A* rs1609682 TT and *HABP2* rs7923349 TT served as independent risk factors for carotid atherosclerosis (OR, 1.45, 95% CI: 1.034–2.032, *p* = 0.031, and OR, 1.829, 95% CI: 1.228–2.723, *p* = 0.003). GMDR analysis indicated that there was a significant gene–gene interaction found among *IL1A* rs1609682, *ITGA2* rs1991013, and *HABP2* rs7923349. After adjusting the covariates, the high-risk interactive genotypes in the 3 variants were significantly associated with a significantly higher risk for carotid atherosclerosis (OR, 2.08, 95% CI: 1.257–5.98, *p* < 0.001).

**Conclusion:**

The prevalence of carotid atherosclerosis was observed to be extremely high in the high-risk stroke population in southwestern China. There were associations observed between the specific variants in inflammation and endothelial function relevant genes and carotid atherosclerosis. The high-risk interactive genotypes among *IL1A* rs1609682, *ITGA2* rs1991013, and *HABP2* rs7923349 significantly increased the risk of carotid atherosclerosis. These results are expected to provide novel strategies for the prevention of carotid atherosclerosis. The gene–gene interactive analysis used in this study may be very helpful to elucidate complex genetic risk factors for carotid atherosclerosis.

## Introduction

Stroke is one of important causes of adult mortality as well as disability in the Western countries and in China (1), and is primarily caused by carotid atherosclerosis (2). The patients with carotid atherosclerosis have a significantly higher risk for stroke and other cardiovascular events as a result of luminal stenosis or plaque rupture (2–4). Carotid atherosclerosis, including carotid plaque, increased intima-media thickness (IMT) and carotid stenosis, is considered as powerful subclinical predictors of the future vascular events (4, 5). Therefore, it is important to investigate the potential etiology of carotid atherosclerosis for better prevention of stroke and other vascular events. Although the associations between carotid atherosclerosis and traditional vascular risk factors have been reported, current studies have focused on the effect of genetic factors because of their potential contributions to the vascular lesions (6, 7). However, up to date, such a genetic effect on carotid atherosclerosis is not clear.

Atherosclerosis is a chronic immune inflammatory process related to a variety of immune-inflammatory cells and mediators and causes instability of plaque or plaque rupture. The risk of atherosclerosis increases with an increase in plaque vulnerability (8). Atherosclerosis as a complex inflammatory disorder, activation and recruitment of the various inflammatory cells, endothelial injury, smooth muscle cell proliferation, and influx of the lipoproteins through vessel injury space are important mechanisms of atherosclerosis (8, 9). Thus, inflammation and endothelial injury play key roles in the pathogenesis of atherosclerosis. The variable risk for atherosclerosis reflects the variants that can effectively modulate endothelial function and inflammatory response in the arterial walls (9). A number of previous studies have demonstrated that various genes related to inflammation are associated with vulnerability of the carotid plaque (6, 10, 11). Furthermore, variants in the genes related to endothelial function and inflammation have been found to play key function in carotid plaque and carotid stenosis (7, 12, 13). A study from the Northern Manhattan population examined the association between carotid plaque and 197 single nucleotide polymorphisms (SNPs) in 43 genes implicated in inflammation and endothelial function, and found that the associations between variants in 10 genes (*TNF*, *NOS2A*, *IL6R*, *TNFSF4*, *PPARA*, *IL1A*, *TLR4*, *ITGA2, VCAM1,* and *HABP2*) and carotid plaque phenotypes (12). Studies from Chinese population also demonstrated that specific SNPs in inflammation and endothelial function relevant genes were associated with carotid plaque, the high-risk interactive genotype among rs7923349, rs1991013, rs1609682, and rs8081248 was independently associated with a higher risk for vulnerable plaque (7), and the high-risk interaction in *ITGA2* rs4865756 and *HABP2* rs7923349 increased the risk of carotid stenosis (13).

In general, carotid plaque, increased IMT and carotid stenosis can exist simultaneously in patients with carotid atherosclerosis. Thus, it might be inappropriate to analyze the possible association of endothelial function and inflammation relevant genetic SNPs with carotid plaque or carotid stenosis separately. Atherosclerosis is a complex disease and it does not follow Mendelian mode of Inheritance (14), which could be attributed to the effect of gene–gene interactions (6, 7). However, few studies have examined the effect of gene–gene interactions among various genes regulating inflammation and endothelial function on the carotid atherosclerosis.

According to China National Stroke Screening Survey (CNSSS) program (1), we carried out this population-based high-risk stroke population survey in Sichuan of southwestern China (15). On the basis of our survey, we performed this study to investigate: (1) the prevalence of carotid atherosclerosis in the high-risk stroke population; (2) the associations of 19 SNPs in genes relevant to endothelial function and inflammation with carotid atherosclerosis, and the influence of gene–gene interaction among the 19 SNPs on carotid atherosclerosis. Overall, the findings can be very important to identify genetic etiology of carotid atherosclerosis, and can aid in better prevention of atherosclerosis and vascular events.

## Materials and methods

### Study population

This multicenter community-based sectional survey was a part of CNSSS, which was approved by Stroke Screening and Prevention Commission in China (Grant No. 2011BAI08B01) (16). The study protocol was reviewed and approved by the Ethics Committee of Suining Central Hospital, the Affiliated Hospital of Southwest Medical University, and the People’s Hospital of Deyang City. A written informed consents were obtained from all the participants before enrollment.

The implementation and organization of this survey can be found in the articles previously published by our group (15–17). Briefly, the 8 communities in Sichuan were randomly selected during May 2015 to September 2015. The residents aged ≥40 years who lived in the community for more than 6 months were surveyed using the structured face-to-face questionnaire. The questionnaire included details about the demographic characteristics, behavioural factors, family and personal history of stroke, history of chronic diseases (such as diabetes mellitus, hypertension, atrial fibrillation, and dyslipidemia), and physical examination. For the subjects who were identified to be as a high-risk for stroke, carotid ultrasonography was measured.

### Evaluation of risk factors and definitions of high-risk stroke population

The eight different conventional risk factors were evaluated, including overweight/obesity, smoking, physical inactivity, family history of stroke, diabetes mellitus, hypertension, atrial fibrillation, and dyslipidemia. The detailed diagnostic criteria for the eight conventional risk factors have been described in our previous study (17).

The individuals were defined as the high-risk stroke population if they had at least three of aforementioned eight conventional risk factors for stroke, or a history of stroke (15–17). The history of stroke was identified by self-reporting and the neuroimaging (magnetic resonance imaging or brain computed tomography scan) (15). Exclusion criteria included: (1) subjects declined to participate in this study; (2) severe cardiovascular, liver or renal disease; (3) hematological diseases, acute or chronic inflammation, immune system diseases, and malignant tumors; (4) history of carotid artery stenting or endarterectomy.

### Data cleaning procedures

**Figure 1:**
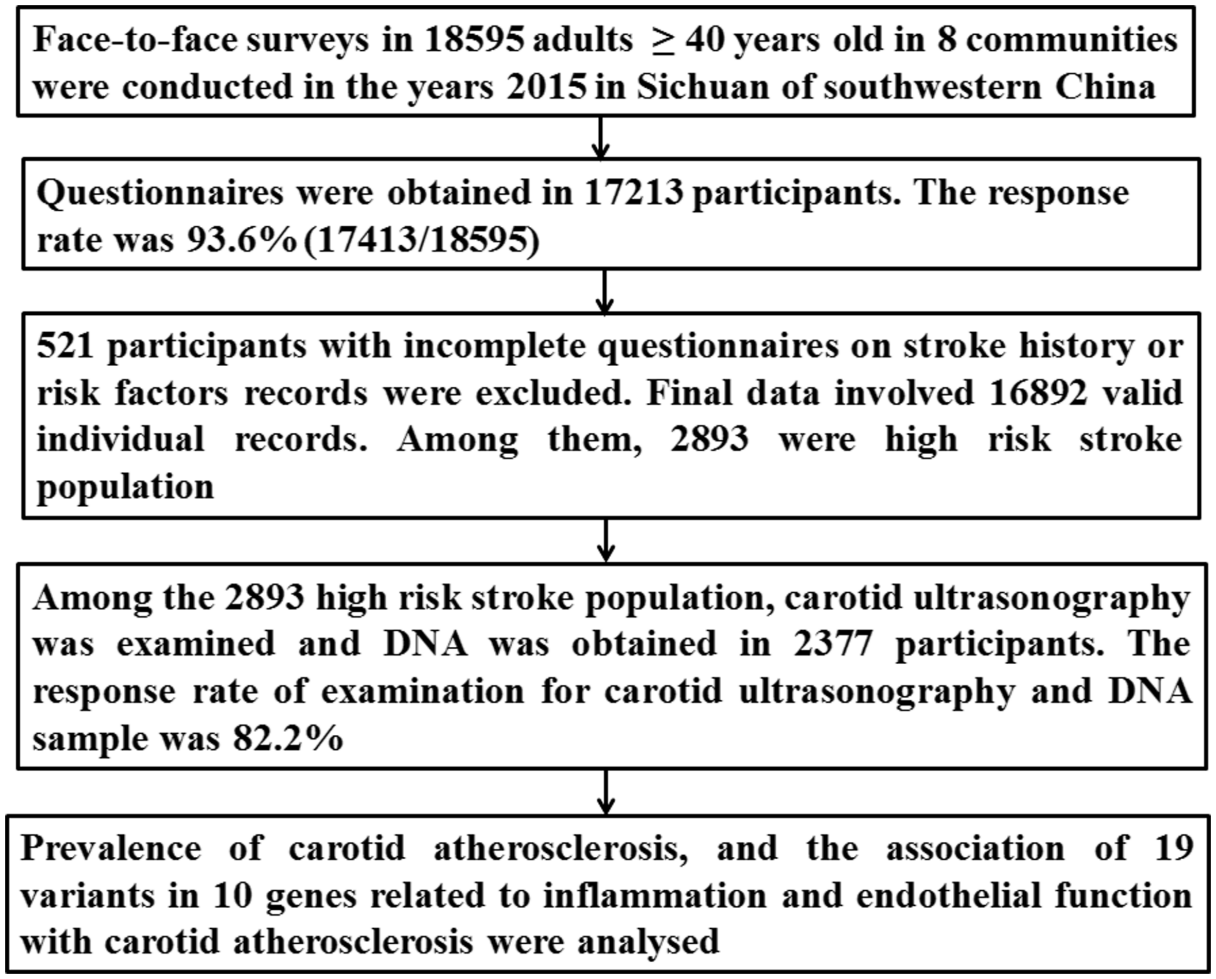
Flow chart in this study.

The detailed procedure has been presented in Figure 1. Briefly, 2,893 individuals were identified as the high-risk stroke population among 16,892 participants. Both DNA and carotid ultrasonography information was obtained in the 2,377 subjects among the 2,893 high-risk stroke population.

### Carotid ultrasonography and definition of carotid atherosclerosis

**Figure 2:**
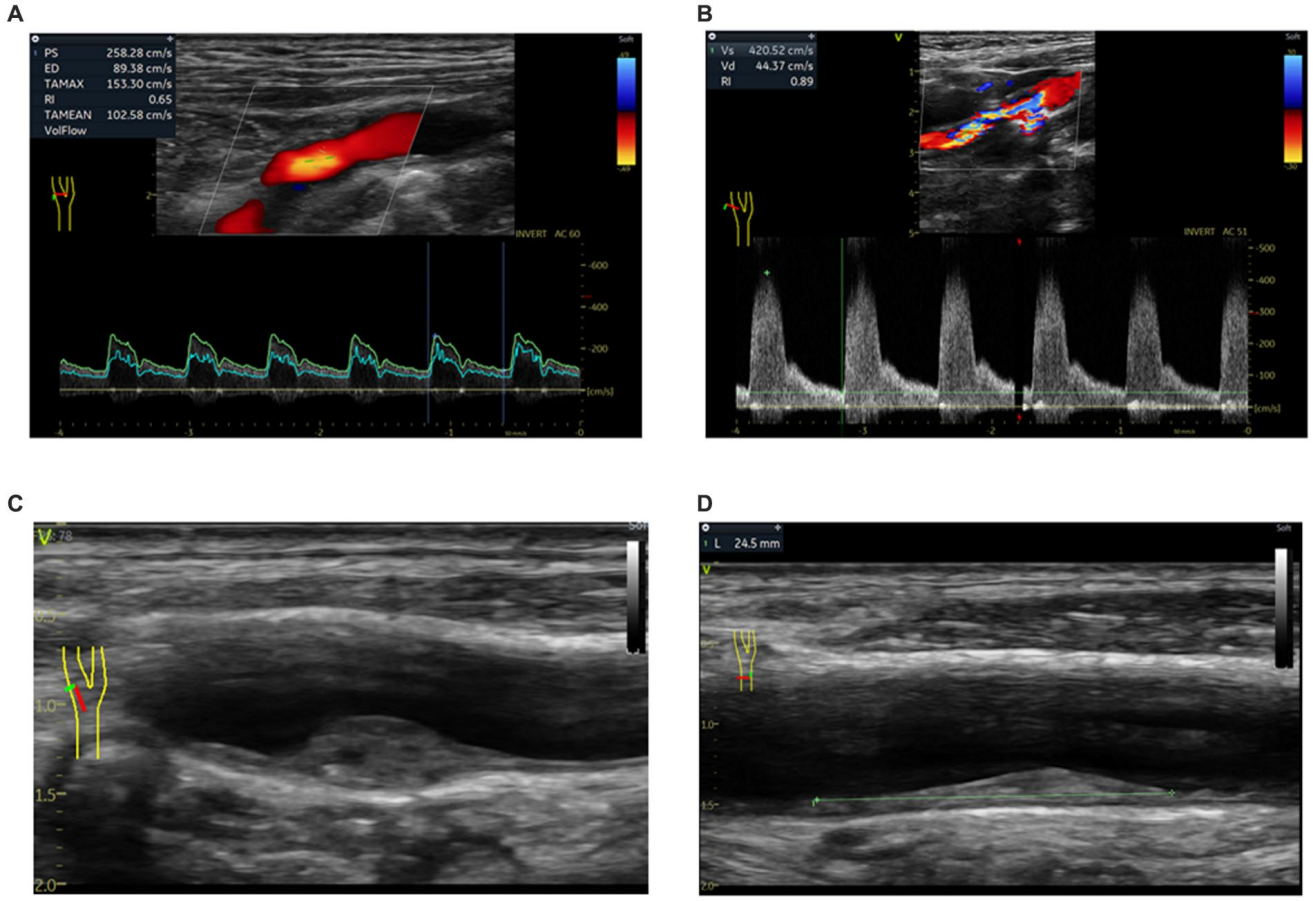
Characteristics of carotid atherosclerosis by carotid ultrasonography. **(A)** >50–69% stenosis internal carotid artery; **(B)** > 70% stenosis internal carotid artery; **(C)** vulnerable plaque in bifurcation; **(D)** stable plaque in common carotid artery.

Bilateral common and internal carotid arteries and bifurcations were evaluated using the Color duplex scan (Acuson Sequoia Apparatus, type 512, 7.5-MHz probe, Berlin, Germany) in 2377 high-risk stroke population, according to the standard scanning and reading protocols (5, 7, 13). The common markers for carotid characteristics, including IMT, plaque and extracranial carotid stenosis were measured. The detailed procedure and definition for carotid plaques, degree of carotid stenosis, and interobserver and intraobserver coefficients have been described in detail in our previous articles (7, 13). The IMT was evaluated from the intima-lumen interface to the media-adventitia interface in each carotid segment and outside the segment of plaque when the plaque was present in a given segment. The mean of IMT was evaluated at 6 carotid sites: common carotid artery (20 mm from the flow divider), bifurcation, and internal carotid artery (20 mm from the flow divider) bilaterally. Mean IMT > 0.9 mm was considered as abnormal (18). The carotid arteries were assessed by ultrasound investigators blinded to the clinical data. Carotid atherosclerosis was defined as the presence of any carotid plaque or any carotid stenosis ≥15% or Mean IMT > 0.9 mm (19) (Figure 2).

### Genotyping

Nineteen SNPs in 10 genes related to endothelial function and inflammation were obtained from NCBI database^1^ following the criteria: (1) the 19 variants have been assessed in the previous studies (7, 12); (2) with the minor allele frequency > 0.05 in each SNP; (3) nonsynonymous variants; (4) the variants might lead to amino acid changes; (5) Tagging SNPs across different human populations.^2^

The peripheral blood (3 ml) was drawn from an arm vein, DNA was extracted by modified phenol/chloroform method (6, 7), and genotypes of the 19 SNPs were evaluated by using matrix-assisted laser desorption/ionization time of flight mass spectrometry method, as described previously by us (7, 13). The investigators were blinded to the clinical data of participants.

### Statistical analysis

Statistical analyses were carried out using the SPSS 17.0 (SPSS Inc., New York, United States). Intergroup differences in the baseline characteristics and genotype distributions of the 19 SNPs were evaluated by χ^2^ test or Fisher’s exact test for the categorical variables. Student *t-*test or analysis of variance was used for the continuous variables between individuals with and without carotid atherosclerosis. Hardy–Weinberg equilibrium for allele frequencies was analyzed by χ^2^-test.

Gene–gene interactions among the 19 SNPs were analyzed under the various scenarios using generalized multifactor dimensionality reduction (GMDR) approach (20), as previously described by us (6, 7). In brief, the GMDR computes the maximum likelihood estimates and the scores of all individuals under the null hypothesis. The 19 SNPs were coded from number 1 to 19, a cumulative score was then calculated within each multifactor cell, which was labeled either as high-risk if the average score exceeded a pre-assigned threshold of zero or as low-risk if score was less than zero. An exhaustive search of all possible models was conducted for all SNPs. The model with minimum prediction error, maximum cross-validation consistency score and a *p-*value ≤0.05 [obtained automatically from the sign test in the GMDR software (20)] was defined as the best model. Then the model was confirmed using a permutation test implemented in the GMDR software.

The prevalence of carotid atherosclerosis between individuals with and without high risk interactive genotypes was compared using χ^2^-test. Multivariate logistic regression analysis was performed to evaluate the potential risk for carotid atherosclerosis conferred by the high-risk interactive genotypes, and the odds ratio (OR) with 95% confidence interval (CI) was reported. The other variables that exhibited a significant association with carotid atherosclerosis (*p* < 0.05) in the univariate analysis were introduced into the multivariate logistic regression model. Furthermore, Hosmer and Lemeshow (H-L) test was used to evaluate the goodness of fit of multivariate logistic regression model. All the tests were two sided, and *p-*value <0.05 denoted statistical significance.

## Results

### Prevalence of carotid atherosclerosis in the high-risk population for stroke

Among the 2,377 participants with high risk stroke population, carotid atherosclerosis was present in 1028 subjects (43.2%), among which 852 (35.8%) cases had carotid plaque [454 (53.3%) had stable plaque, 398 (46.7%) had vulnerable plaque], 295 (12.4%) cases had carotid stenosis [244 (82.7%) had 15–49% stenosis, 51 (17.29%) had more than 50% stenosis], whereas 445 (18.7%) had mean IMT > 0.9 mm (Table 1). It was observed that compared with individuals without carotid atherosclerosis, individuals with carotid atherosclerosis were older, had a higher proportion of males, rural residents, with junior middle school or below level of education, and had a history smoking, hypertension and dyslipidemia (*p* < 0.05, Table 1).

**Table 1:** Demographic characteristics of the high-risk population for stroke with and without carotid atherosclerosis [*n*(%)].

Variables	Carotid atherosclerosis (*n* = 1,028)	Non-carotid atherosclerosis (*n* = 1,349)	*p*-Value
Sex			0.004
Male	495 (48.2)	570 (42.3)	
Female	533 (51.8)	779 (57.7)	
Age, y			<0.001
40–49	42 (4.1)	176 (13.0)	
50–59	182 (17.7)	367 (27.2)	
60–69	415 (40.4)	545 (40.4)	
70–79	325 (31.6)	219 (16.2)	
≥80	64 (6.2)	42 (3.1)	
Residence			<0.001
Urban	415 (40.4)	649 (48.1)	
Rural	613 (59.6)	700 (51.9)	
Education			0.001
Junior middle school or below	958 (93.2)	1,205 (89.3)	
Senior middle school or above	70 (6.8)	144 (10.7)	
Overweight/obesity			0.155
Yes	546 (53.1)	756 (56.0)	
No	482 (46.9)	593 (44.0)	
Smoking			<0.001
Yes	413 (40.2)	403 (29.9)	
No	615 (59.8)	946 (70.1)	
Physical inactivity			0.318
Yes	641 (62.4)	868 (64.3)	
No	387 (37.6)	481 (35.7)	
Hypertension			<0.001
Yes	820 (79.8)	983 (72.9)	
No	208 (20.2)	366 (27.1)	
Diabetes			0.739
Yes	286 (27.8)	367 (27.2)	
No	742 (72.2)	982 (72.8)	
Dyslipidemia			
Yes	319 (31.0)	456 (33.8)	0.153
No	709 (69.0)	893 (66.2)	
Atrial fibrillation			0.296
Yes	18 (1.8)	32 (2.4)	
No	1,010 (98.2)	1,317 (97.6)	
Family history			0.192
Yes	173 (16.8)	255 (18.9)	
No	855 (83.2)	1,094 (81.1)	
History of stroke			0.522
Yes	186 (18.1)	258 (19.1)	
No	842 (81.9)	1,091 (80.9)	
Carotid plaque	852 (35.8)	--	
Stable plaque	454 (19.1)	--	
Vulnerable plaque	398 (16.7)	--	
Carotid stenosis		--	
15–49% stenosis	244 (10.3)	--	
**≥**50% stenosis	51 (2.1)	--	
Mean IMT ≥1.0 mm	445 (18.7)	--	

### Distribution of genotypes in the subjects

The genotype distributions of the 19 variants analyzed in this study were in agreement with Hardy–Weinberg Equilibrium (*p* > 0.05). Moreover, univariate analyses showed that there were significant differences in genotype distributions of *IL1A* rs1609682, *PPARA* rs4253655, and *HABP2* rs7923349 between individuals with and without carotid atherosclerosis (*p* < 0.05, Table 2).

**Table 2:** Genotype distribution in individuals with and without carotid atherosclerosis (%).

	Carotid atherosclerosis (*n* = 1,028)	Non- carotid atherosclerosis (*n* = 1,349)	Wald χ2 value	*P-*value
*IL6R* (rs4845625)			3.052	0.217
TT	297 (28.9)	358 (26.5)		
CC	222 (21.2)	328 (24.3)		
CT	509 (49.4)	663 (49.1)		
*IL6R* (rs1386821)			2.158	0.394
GT	69 (6.7)	112 (8.3)		
GG	3 (0.3)	4 (0.3)		
TT	956 (93.0)	1,233 (91.4)		
*IL1A* (rs1800587)			1.963	0.370
AG	141 (13.7)	173 (12.8)		
GG	883 (85.9)	1,165 (86.4)		
AA	4 (0.4)	11 (0.8)		
*IL1A* (rs1609682)			9.072	0.011
GG	457 (44.5)	576 (42.7)		
GT	479 (46.6)	690 (51.1)		
TT	92 (8.9)	83 (6.2)		
*PPARA* (rs4253778)			0.006	0.938
CG	2 (0.2)	4 (0.3)		
GG	1,026 (99.8)	1,345 (99.7)		
*PPARA* (rs4253655)			5.258	0.035
AG	4 (0.4)	0 (0.0)		
GG	1,024 (99.6)	1,349 (100.0)		
*TLR4* (rs752998)			2.589	0.274
TT	19 (1.8)	36 (2.7)		
GG	733 (71.3)	931 (69.0)		
GT	276 (26.8)	382 (28.3)		
*TLR4* (rs1927911)			1.203	0.58
AG	509 (49.5)	650 (48.2)		
AA	163 (15.9)	203 (15.0)		
GG	356 (34.4)	496 (36.8)		
*TNFSF4* (rs1234313)			4.691	0.096
AG	487 (47.4)	579 (42.9)		
GG	116 (11.3)	163 (12.1)		
AA	425 (41.3)	607 (45.0)		
*TNFSF4* (rs11811788)			0.200	0.905
CG	158 (15.4)	215 (15.9)		
GG	11 (1.1)	13 (1.0)		
CC	859 (83.6)	1,121 (83.1)		
*NOS2A* (rs8081248)			0.447	0.800
AG	459 (44.6)	591 (43.8)		
AA	106 (10.3)	150 (11.1)		
GG	463 (45.0)	608(45.1)		
*NOS2A* (rs2297518)			0.151	0.927
AG	277 (26.9)	373 (27.7)		
AA	22 (2.1)	28 (2.1)		
GG	729 (70.9)	948 (70.3)		
*TNF* (rs3093662)			0.485	0.486
AG	52 (5.1)	60 (4.4)		
AA	976 (94.9)	1,289 (95.6)		
*VCAM1*(rs2392221)			3.407	0.182
CT	252 (24.5)	293 (21.7)		
CC	756 (73.5)	1,021 (75.7)		
TT	20 (1.9)	35 (2.6)		
*VCAM1* (rs3783615)				Na
AA	1,028 (100.0)	1,349 (100.0)		
*HABP2* (rs7923349)			12.141	0.002
TT	68 (6.6)	49 (3.6)		
GT	389 (37.8)	499 (37.0)		
GG	571 (55.5)	801 (59.4)		
*HABP2* (rs932650)			1.588	0.452
CT	452 (44.0)	578 (42.8)		
CC	91 (8.9)	140 (10.4)		
TT	485 (47.2)	631 (46.8)		
*ITGA2* (rs1991013)			2.056	0.358
GG	90 (8.8)	141 (10.5)		
AA	480 (46.7)	628 (46.6)		
AG	458 (44.6)	580 (43.0)		
*ITGA2* (rs4865756)			2.531	0.282
AG	401 (39.0)	488 (36.2)		
GG	557 (54.2)	775 (57.4)		
AA	70 (6.8)	86 (6.4)		

### Gene–gene interactions among the 19 variants

The association of gene–gene high-order interaction in the 19 variants with carotid atherosclerosis was evaluated using the GMDR approach. A significant gene–gene interaction was found in the 19 variants, and the best interactive model for carotid atherosclerosis was interaction among *IL1A* rs1609682, *ITGA2* rs1991013 and *HABP2* rs7923349, which scored 10/10 for the cross-validation consistency and 10 for sign test (*p* = 0.001, Table 3). The *p-*value of prediction error was 0.016 for the GMDR based on the permutation testing.

**Table 3:** GMDR analysis of the best models, prediction accuracies, cross-validation consistencies, and *p-*values for carotid atherosclerosis.

Best model*	Training balanced accuracy	Testing balanced accuracy	Cross- validation consistency	Sign test (*P-*value)
1	0.562	0.531	8/10	5 (0.214)
1, 2	0.543	0.527	7/10	7 (0.053)
1, 2, 3	0.572	0.526	10/10	10 (0.001)
1, 2, 3, 4	0.593	0.576	7/10	9 (0.056)
1, 2, 3, 4, 5	0.592	0.586	8/10	5 (0.367)
1, 2, 3, 4, 5, 6	0.684	0.633	6/10	8 (0.339)
1, 2, 3, 4, 5, 6, 7	0.635	0.612	9/10	6 (0.136)
1, 2, 3, 4, 5, 6, 7, 8	0.632	0.529	7/10	7 (0.292)
1, 2, 3, 4, 5, 6, 7, 8, 9	0.723	0.712	5/10	8 (0.623)
1, 2, 3, 4, 5, 6, 7, 8, 9, 10 1, 2, 3, 4, 5, 6, 7, 8, 9, 10, 11	0.598 0.537	0.562 0.387	10/10 6/10	10 (0.552) 9 (0.182)
1, 2, 3, 4, 5, 6, 7, 8, 9, 10, 11, 12	0.586	0.575	7/10	5 (0.372)
1, 2,3, 4, 5, 6, 7, 8, 9, 10, 11, 12, 13	0.637	0.625	7/10	7 (0.089)
1, 2, 3, 4, 5, 6, 7, 8, 9, 10, 11, 12, 13, 14	0.588	0.523	6/10	8 (0.562)
1, 2, 3, 4, 5, 6, 7, 8, 9, 10, 11, 12, 13, 14, 15	0.629	0.627	7/10	8 (0.394)
1, 2, 3, 4, 5, 6, 7, 8, 9, 10, 11, 12, 13, 14, 15, 16	0.625	0.608	8/10	9 (0.288)
1, 2, 3, 4, 5, 6, 7, 8, 9, 10, 11, 12, 13, 14, 15, 16, 17	0.514	0.497	6/10	10 (0.593)
1, 2, 3, 4, 5, 6, 7, 8, 9, 10, 11, 12, 13, 14, 15, 16, 17, 18	0.553	0.512	10/10	6 (0.264)
1, 2, 3, 4, 5, 6, 7, 8, 9, 10, 11, 12, 13, 14, 15, 16, 17, 18, 19	0.645	0.623	8/10	7 (0.396)

GMDR, generalized multifactor dimensionality reduction. *Numbers 1–19 represent rs1609682, rs7923349, rs1991013, rs8081248, rs4253778, rs2297518, rs4253655，rs3093662, rs4845625, rs1386821, rs1234313, rs1800587, rs3783615, rs2392221, rs11811788, rs752998, rs1927911, rs4865756, and rs932650, respectively.

### Different genotype combinations with the risk of carotid atherosclerosis

Thereafter, the associations of different genotype combinations in the 3 three interactive variants with the risk of carotid atherosclerosis were evaluated. It was found that compared with the individuals with wild-type genotype of the three variants (i.e., rs1609682 GG, rs1991013 GG, and rs7923349 GG), the relative risk of the different genotype combinations in the three variants for carotid atherosclerosis was assessed. The results showed that the 4 genotype combinations contributed to the larger risk for carotid atherosclerosis, including those subjects carrying rs1609682 TT, rs1991013 AA and rs7923349 TT (OR = 2.62, 95% CI: 1.21–6.86, *p* = 0.007); rs1609682 TT, rs1991013 AG and rs7923349 TT (OR = 2.06, 95% CI: 1.07–4.62, *p* = 0.036); rs1609682 GT, rs1991013 AA and rs7923349 GT (OR = 1.76, 95% CI: 1.03–2.63, *p* = 0.043); rs1609682 TT, rs1991013 AA and rs7923349 GT (OR = 2.14, 95% CI: 1.18–5.37, *p* = 0.012) (Table 4), which were considered as the high-risk interactive genotypes. The other combinations among the three variants did not achieve statistical significance (*p* > 0.05) and were thus considered as the low-risk interactive genotypes.

**Table 4:** Associations between genotype combinations and the risk of carotid atherosclerosis.

rs1609682	GG	TT	TT	TT	GT	TT, GT	TT	TT, GT
rs7923349	GG	TT	GT	TT	GT	TT	TT, GT	TT, GT
rs1991013	GG	AA	AA	AG	AA	AA, AG	AA	AA, AG
OR	1*	2.62	2.14	2.06	1.76	1.27	1.45	1.12
95% CI	–	1.21–6.86	1.18–5.37	1.07–4.62	1.03–2.63	0.92–1.69	0.82–2.23	0.78–1.69
*P-*value	–	0.007	0.012	0.036	0.043	0.224	0.621	0.579

*The wild-type genotype for each genetic factor was used as the reference OR. OR, odds ratio; CI, confidence interval.

### Association of the high-risk interactions with carotid atherosclerosis

There were 523 subjects identified who were carrying the high-risk interactive genotypes in the 2,377 high risk stroke population. The prevalence of carotid atherosclerosis was found to be significantly higher in the subjects carrying the high-risk interactive genotypes in comparison to those carrying the low-risk interactive genotypes (56.6% [296/523] vs. 39.5% [732/1854], χ^2^ = 48.68, *p* < 0.001).

Furthermore, multivariate logistic regression was employed to evaluate the risk of carotid atherosclerosis conferred by the high-risk interactive genotypes among *IL1A* rs1609682, *HABP2* rs7923349, and *ITGA2* rs1991013. The low-risk interactive genotypes were assigned as zero, whereas the high-risk interactive genotypes were designated as one. The other variables that showed a significant association with carotid atherosclerosis (*p* < 0.05) in the univariate analysis were entered the multivariate logistic regression model to adjust. The results exhibited that the high-risk interactive genotypes in *IL1A* rs1609682, *ITGA2* rs1991013, and *HABP2* rs7923349 were independently associated with a higher risk for carotid atherosclerosis after adjusting covariates (OR, 2.08, 95% CI: 1.257–5.980, *P* < 0.001, Table 5). Furthermore, H-L test was used to evaluate the goodness of fit of the multivariate logistic regression model, and the result showed that the goodness of fit of the model was well (χ^2^ value = 4.324, *p* = 0.823).

**Table 5:** Multivariate analysis of the major risk factors for carotid atherosclerosis.

Risk factor	OR*	95% CI	*P-*value
Age	1.053	1.043–1.063	<0.001
Male	1.093	0.880–1.358	0.421
Rural	1.274	1.071–1.515	0.006
Junior middle school or below	1.118	0.810–1.543	0.498
Smoking	1.544	1.233–1.933	<0.001
Hypertension	1.252	1.018–1.539	0.033
*IL1A* rs1609682 TT	1.450	1.034–2.032	0.031
*PPARA* rs4253655 AG	1.122	0.892–1.686	0.253
*HABP2* rs7923349 TT	1.829	1.228–2.723	0.003
High-risk interactive genotypes	2.08	1.257–5.980	<0.001

OR, odds ratios; CI, confidence interval.

## Discussion

In the present study, we have observed a high prevalence of carotid atherosclerosis (43.2% [1,028/2377)] in the high-risk stroke population and the associations of variants in two distinct genes related to inflammation (*IL1A* rs1609682, *PPARA* rs4253655) and one gene related to endothelial function (*HABP2* rs7923349) with carotid atherosclerosis in southwestern China. Furthermore, statistical interactions were found between three SNPs (*IL1A* rs1609682, *HABP2* rs7923349 and *ITGA2* rs1991013) and carotid atherosclerosis by GMDR analysis, and the high-risk interactive genotypes in the three SNPs were significantly associated with a higher risk for carotid atherosclerosis.

Numerous studies have previously demonstrated that carotid atherosclerosis (including carotid plaque, increased IMT, and carotid stenosis) can be considered as subclinical predictors of the future vascular events (4, 5). In this study, we found that prevalence of carotid atherosclerosis was high (43.2%) in the high-risk stroke population, and old age, smoking and hypertension were identified as potential risk factors for carotid atherosclerosis. Our results were in agreement with other prior studies (5, 21). Hypertension is a very important risk factor for carotid atherosclerosis and stroke (21). However, the proportion of people whose hypertension is controlled is extremely low in China (17, 22). In this survey, only 40.0% (950/2377) of patients with hypertension were receiving optimal antihypertensive treatment. Smoking contributed to an increased risk of carotid atherosclerosis, and smoking prevalence has consistently increased in the past three decades in China (23). Thus, behavioral interventions for smoking and clinical control of hypertension could be useful for preventing carotid atherosclerosis.

A number of previous studies have also explored the associations of inflammation and endothelial function relevant genetic SNPs with stroke (24, 25), but only few studies have primarily focused on the subclinical carotid atherosclerosis. For instance, Gardener et al. (12) was the first to evaluated the associations of variants in the genes related to endothelial function and inflammation with carotid plaque in Hispanics from Northern Manhattan, and they demonstrated that variants in 10 different genes linked with inflammation and endothelial function (*NOS2A*, *TNF*, *IL6R*, *PPARA*, *TNFSF4*, *TLR4*, *IL1A*, *ITGA2*, *HABP2,* and *VCAM1*) were associated the carotid plaque phenotypes. Our previous studies have also revealed significant associations of variants in genes related to endothelial function and inflammation with vulnerable carotid plaque (7) and carotid stenosis (13) in the Chinese population. However, carotid plaque, increased IMT, and carotid stenosis were present simultaneously in patients with carotid atherosclerosis. Thus, it may be inappropriate to analyze the associations of the genetic variants with carotid plaque, increased IMT or carotid stenosis separately. According to findings of the previous studies (18, 21), carotid atherosclerosis was defined as presence of any carotid plaque or any carotid stenosis ≥15% or mean IMT > 0.9 mm in this study, and we found that there were significant associations between the polymorphisms of *IL1A* rs1609682, *PPARA* rs4253655, and *HABP2* rs7923349 and carotid atherosclerosis.

Interleukin-1 (IL-1) as a cytokine play a key role in “response to injury” model of atherosclerosis and stroke (26). In animal models, IL-1a was found to be up-regulated after acute middle cerebral artery occlusion, inducing migration, proliferation and angiogenesis in brain endothelial cells (27). After cerebral ischemia, IL-1a express in microglia, astrocytes, and endothelial cells, inducing activation of astrocytes and endothelial cells and promoting formation of tube-like structure that is an important hallmark of angiogenesis, knockout IL-1a in mice can reduce ischemic damage (27, 28). Polymorphisms in *IL1A* gene may increase the risk of ischemic stroke (29). In this study, we also revealed polymorphisms in *IL1A* were associated with carotid atherosclerosis. SNPs in inflammatory genes can interact with the conventional risk factors directly or indirectly to influence the development of atherosclerosis. IL-1α in macrophage is an important cytokine regulating the development of atherosclerosis (30), over-representation of *IL1A* gene was associated with the coronary artery disease (31). In addition, prior studies also support the association between variant in *IL1A* gene and carotid atherosclerosis (7, 12, 32). Polymorphisms in *IL1A* can significantly increase the susceptibility for carotid atherosclerosis, and the *IL1A* allele 2 might influence the inflammatory environment in the vascular endothelium (33). Peroxisome proliferator-activated receptor alpha (PPARα) as a nuclear receptor when activated, can effectively trigger the acyl-CoA oxidase transcription by catalyzing the fatty acid *β*-oxidation pathway. Polymorphisms in *PPARA* gene have been demonstrated to substantially affect the oxidative stress, lipid metabolism, progression of coronary atherosclerosis, and the risk of myocardial infarction (34, 35). However, studies on the potential relationship between *PPARA* SNPs and carotid atherosclerosis are lacking in the literature. *PPARA* rs4253655 SNPs have been associated with carotid plaque and vulnerable plaque in a cohort of Northern Manhattan (12) and a community-based study of China (7). In present study, we found that *PPARA* rs4253655 was also associated with carotid atherosclerosis, thereby indicating its key role in the different stages of carotid atherosclerosis.

The endothelial function maintains the vascular barrier by controlling platelet adhesion and aggregation, platelet and immune cell interactions, capillary tone and interendothelial cell adherence. Endothelial dysfunction may damage vascular integrity, is associated with various human diseases such as stroke, atherosclerosis, and coronary artery disease (36). Vascular integrity is regulated by hyaluronan-binding protein 2 *(HABP2)* gene, which encodes a cell adhesion protein (hyaluronan-binding protein 2). *HABP2* gene may be a genetic susceptibility locus in stroke (25). HABP2 has been shown to effect the vascular smooth muscle cell proliferation and modulate the vulnerability of atherosclerotic plaque (37). Our previous studies as well as other prior reports have demonstrated that *HABP2* rs7923349 variants were associated with the carotid stenosis (13, 38), and carotid plaque (7, 12). Here, we found that *HABP2* rs7923349 was also associated with carotid atherosclerosis. Gardener et al. (12) reported the associations between variants in *NOS2A, TNF, IL6R, TLR4, TNFSF4,* and *VCAM1* genes and carotid atherosclerosis. However, in this study we found that the genotypes of these genes were irrelevant to carotid atherosclerosis. The differences in the findings could be attributed to the different study population, as gene SNPs can vary greatly among the different ethnic population.

Atherosclerosis is a complex disease, as it does not follow the Mendelian mode of inheritance, which may be the result of gene–gene interactions (6, 7). Single gene approach may not be effective to find the genetic etiology of the complex disease, and it has been emphasized that assessment of gene–gene interactions is necessary to investigate the genetic mechanisms for the complex diseases, such as carotid atherosclerosis. Interestingly, in a previous study Gardener et al. (12) revealed there were interactions among the haplotypes in *IL6R, TNFSF4, NOS2A* and *PPARA* for thick plaque, and interactions between the haplotypes in *PPARA* and *IL1A* for irregular plaque. Our previous studies have also found that the high-risk interactions among *HABP2* rs7923349, *IL1A* rs1609682 *ITGA2* rs1991013 and *NOS2A* rs8081248 increased the risk for the carotid vulnerable plaque (7), and potential interactions between *ITGA2* rs4865756 and *HABP2* rs7923349 were considered as the risk for carotid stenosis (13). The noteworthy finding in the present study was that there was a significant gene–gene interaction observed in *IL1A* rs1609682, *ITGA2* rs1991013, and *HABP2* rs7923349 using the GMDR approach, and the high-risk interactive genotypes in the three variants were independently risk factors of carotid atherosclerosis. However, the molecular mechanisms of interactions in the three variants are unclear. Integrin alpha 2 (ITGA2) can regulate the cell adhesion and cell-surface-mediated signaling. SNPs of *ITGA2* C807T were associated with carotid IMT, plaque, and the risk of ischemic stroke in patients with type 2 diabetes (10, 39). *ITGA2* rs1991013 was associated with carotid calcified plaque and increased risk of general atherosclerosis (12). A number of previous studies have revealed that the associations of SNPs in *ITGA2*, *IL1A, HABP2* and *NOS2A* genes with inflammation and endothelial function (7, 10, 12, 13, 39). Therefore, one possible explanation is that the three variants can encode and regulate endothelial function and inflammation relevant enzymes, which can participate in the important pathogenic mechanisms for atherosclerosis. However, further studies are needed to explore the molecular mechanisms of interaction among the three variants are necessary in future.

The major strengths of this study include: (1) systematic examination of 19 variants in 10 different genes involved in endothelial function and inflammation; (2) multicenter population-based cross-sectional survey and focus on the high-risk population for stroke; (3) use of the GMDR approach to analyze gene–gene interactions among the 19 SNPs. However, there are also several limitations associated with this this study. First, this was a population-based cross-sectional survey with self-reported questionnaire, and thus, there may be significant recall bias. Second, carotid arteries were only evaluated by ultrasound analysis in this study. Computed tomography angiography and high-resolution magnetic resonance imaging might provide additional information on carotid atherosclerosis. Third, the main aim of present study was to investigate the associations of the 19 SNPs with carotid atherosclerosis, but analysis of the associations of these SNPs was not stratified based on the carotid plaque, carotid stenosis and IMT. Fourth, we investigated the role of several known genes related to inflammation and endothelial function, but the possible involvement of other relevant genes was not assessed. In addition, although we found that the high-risk interactions among *IL1A* rs1609682, *ITGA2* rs1991013 and *HABP2* rs7923349 increased the risk of carotid atherosclerosis, the detailed molecular mechanisms were not investigated. Furthermore, statins, antiplatelet drugs and antihypertensives may affect carotid atherosclerosis, but the effect of these drugs was not examined on carotid atherosclerosis. Finally, as is known to all, intracranial stenosis is more prevalent than extra cranial stenosis in Asian population. However, this multicenter community-based sectional survey was a part of CNSSS program, intracranial stenosis was not evaluated. Thus, we did not know the prevalence of intracranial atherosclerosis in the high-risk stroke population in China.

## Conclusion

In the present study, we found a high prevalence of carotid atherosclerosis in the high-risk stroke population in China and also identified the associations of variants in *IL1A* rs1609682, *PPARA* rs4253655, and *HABP2* rs7923349 with carotid atherosclerosis. There was a significant gene–gene interaction observed in *IL1A* rs1609682, *ITGA2* rs1991013, and *HABP2* rs7923349, the high-risk interactive genotypes in the three variants served as independent risk factors of carotid atherosclerosis. These results are expected to provide novel strategies for prevention of carotid atherosclerosis in Chines population. Based on our findings, active intervention of conventional risk factors, such as hypertension and smoking, may be very important in reducing the risk for stroke in the high-risk stroke population carrying the high-risk interactive genotypes. Furthermore, our findings are expected to identify new gene targets for prevention and treatment of carotid atherosclerosis and stroke, and provide a theoretical basis for drug development and gene therapy against new gene targets in the future. The gene–gene interactive analysis used in present study may be very helpful to elucidate complex genetic risk factors for carotid atherosclerosis.

## Data availability statement

The original contributions presented in the study are included in the article/supplementary material, further inquiries can be directed to the corresponding author.

## Ethics statement

The studies involving human participants were reviewed and approved by the Ethics Committees of Suining Central Hospital, the Affiliated Hospital of Southwest Medical University and the People’s Hospital of Deyang City. Written informed consent to participate in this study was provided by the patients/participants or patients/participants' legal guardian/next of kin.

## Author contributions

YX, XY, and HL designed this study and acquired the funding. HL, MY, TQ, and WW performed the face to face survey and follow up. YX, TQ, and MS drafted the figures and analyzed the results. YX, XY, and MS drafted the manuscript and the tables. XY, HL, and MY supervised this project. All authors contributed to the article and approved the submitted version.

## Funding

This study was supported in part by grants from the Scientific Research Foundation of Sichuan Provincial Health Department (Grant No. 16ZD046). The funding body did not participate in the design of the study; collection, analysis, and interpretation of data; and in writing the manuscript.

## Conflict of interest

The authors declare that the research was conducted in the absence of any commercial or financial relationships that could be construed as a potential conflict of interest.

## Publisher’s note

All claims expressed in this article are solely those of the authors and do not necessarily represent those of their affiliated organizations, or those of the publisher, the editors and the reviewers. Any product that may be evaluated in this article, or claim that may be made by its manufacturer, is not guaranteed or endorsed by the publisher.

## Footnotes

^1^http://www.ncbi.nlm.nih.gov/SNP

^2^http://pga.gs.washington.edu
